# Insights about stabilization of sulforaphane through microencapsulation

**DOI:** 10.1016/j.heliyon.2019.e02951

**Published:** 2019-11-28

**Authors:** Víctor Zambrano, Rubén Bustos, Andrea Mahn

**Affiliations:** Department of Chemical Engineering, University of Santiago of Chile, Avenida Libertador Bernardo O'Higgins, 3363, Santiago, Chile

**Keywords:** Engineering, Chemistry, Food science, Sulforaphane, Encapsulation efficiency, Stability, Degradation kinetics

## Abstract

The health–promoting properties of sulforaphane (SFN) are well known, however its instability is still a hurdle for its incorporation into food matrices. SFN can be stabilized by microencapsulation, technique sparingly explored for isothiocyanates so far. This review summarizes the advances in microencapsulation of SFN and other isothiocyanates. Encapsulation efficiency and degradation rate of sulforaphane in different systems are compared and discussed. Ionic gelation and complex coacervation seem more adequate for SFN, both underexplored until now. Drying conditions after chemical encapsulation are determinant, most likely related to thermal degradation of SFN. The current information is insufficient to identify the most adequate encapsulation system and the optimal process conditions to stabilize SFN aiming at its incorporation into food matrices. Accordingly, encapsulation conditions should be investigated, which arises as a new research line. Stability studies are encouraged since this information will help in designing SFN microencapsulation strategies that extend the industrial application of this promising health-promoting compound.

## 1 Introduction

Sulforaphane (SFN) is an isothiocyanate (ITC) that comes from the hydrolysis of glucoraphanin, the most abundant glucosinolate found in broccoli, and also present in other *Brassicaceae*. Several *in vitro* and *in vivo* studies suggested that sulforaphane may prevent different types of cancer, such as breast cancer (Pawlik et al., 2017), oral cancer (Bauman et al., 2016), colorectal cancer (Chen et al., 2012), prostate cancer (Wong et al., 2014), melanoma (Rudolf et al., 2014) and leukemia (Lin et al., 2012). In addition, sulforaphane has the ability to improve hypertensive states (Senanayake et al., 2012), to prevent type 2 diabetes – induced cardiomyopathy (Zhang et al., 2014) and to protect against gastric ulcer (Zeren et al., 2016). Recently, Shiina et al. (2015) reported that SFN may help in the schizophrenia treatment, and Martins et al. (2018) proposed that SFN has potential to struggle obesity.

Even though sulforaphane has great potential as health-promoting compound, its industrialization has been limited because of its relative instability (Van Eylen, Oey, Hendrickx & Van Loey, 2007). SFN is affected by pH, temperature, heat, light and oxygen. Then its stabilization becomes a technological challenge. An option to stabilize SFN and even increase its bioavailability in a food matrix is microencapsulation. SFN and ITC microencapsulation has been poorly studied. The available studies include microencapsulation by physical (spray-drying, freeze-drying) or chemical (coacervation, emulsification, inclusion complexation, and ionic gelation) methods, and combinations of them. This review focuses on the microencapsulation of SFN and other isothiocyanates and the effect of the microencapsulation technique on the stability and retention of the compound.

## 2 Microencapsulation of sulforaphane and isothiocyanates

### 2.1 General aspects

Encapsulation refers to a process which entraps a substance (active agent) into another substance that conforms the wall material resulting in particles in the nanometer (nanoencapsulation), micrometer (microencapsulation) or millimeter scale. The encapsulated substance corresponds to the “core” while the substance that surrounds the core corresponds to the “wall material”, “encapsulating agent”, “coating”, “membrane”, “shell”, “capsule”, “carrier material”, “external phase” or “matrix” (Fang and Bhandari, 2010).

**Figure 1:**
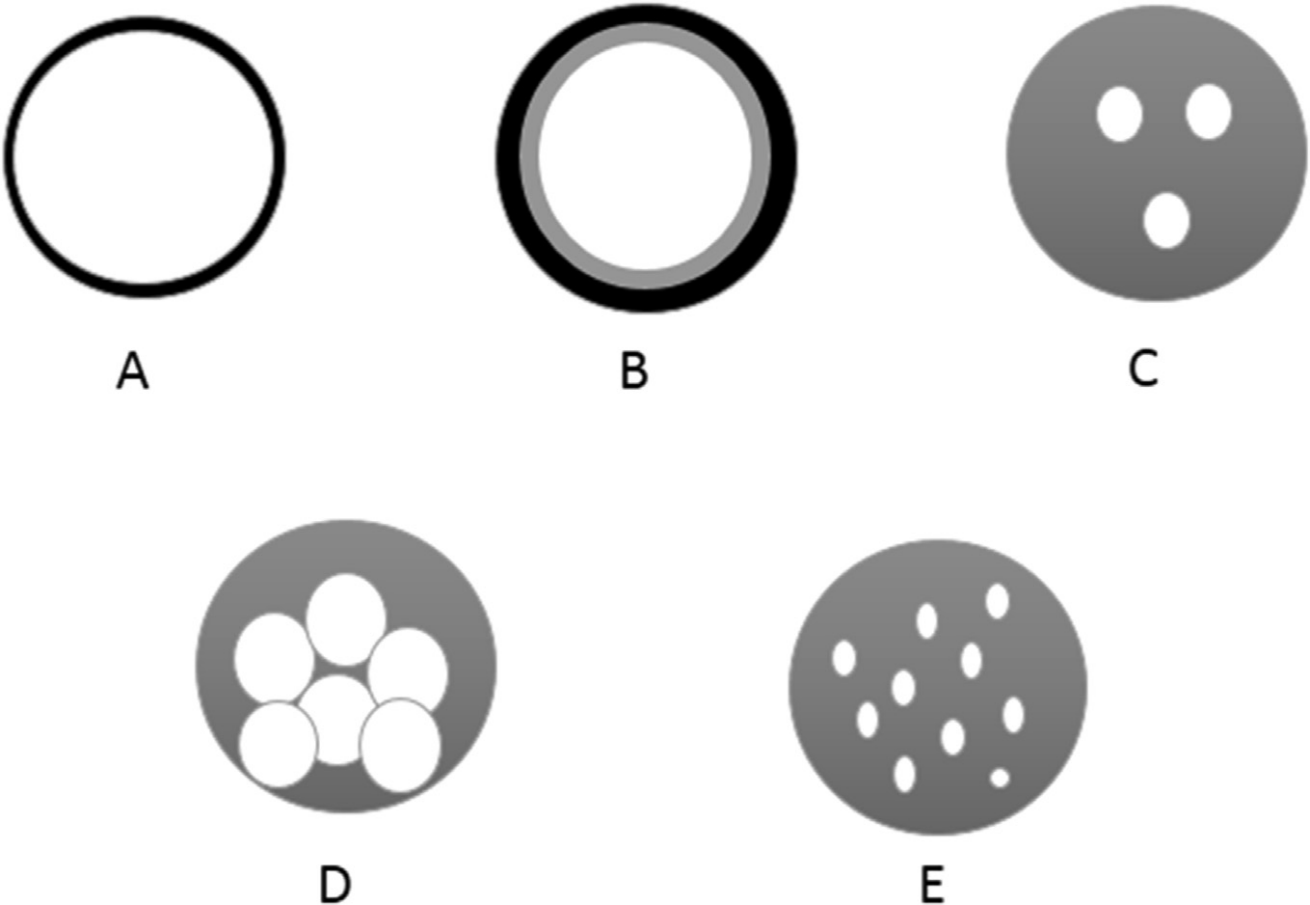
Microcapsules morphology: (A) Simple, (B) Multiwall, (C) Multi-core, (E) Aggregate and (E) Matrix (Adapted from Mishra, 2016).

Microencapsulation is intended to protect bioactive compounds from undergoing undesirable reactions while enhancing their functionality and bioavailability (Lakkis, 2016). The performance of microencapsulation depends on the physical and chemical properties of the material to be microencapsulated. The core material determines to a great extent the morphology of the microcapsules. The morphology of the internal structure of a microcapsule depends on the wall materials and the microencapsulation method. Microcapsules can be mononuclear with multiple shells (such as layering of shells), or they may form clusters of microcapsules (Figure 1) (Mishra, 2016).

Isothiocyanates, and especially sulforaphane, are poorly water-soluble compounds. Given the relative instability of sulforaphane, microencapsulation is a promising approach for its stabilization and improvement of its bioavailability, since this technique protects hydrophobic compounds from degradation associated to external chemical conditions (Rezaei et al., 2019). Table 1 shows the encapsulation efficiency of SFN and ITC through different microencapsulation methods.

**Table 1:** Studies of encapsulation efficiency of sulforaphane and other isothiocyanates.

Active Compound	Chemical encapsulation	Wall materials	Drying	EE (%)	References
Sulforaphane	Complex coacervation	Gelatin/Gum arabic	Vacuum oven	12.2 ± 0.1	García-Saldaña et al. (2016)
		Gelatin/Pectin		17.9 ± 1.3	
Sulforaphane	Simple coacervation	Maltodextrin (MD)	Spray drying	39.1 ± 2.6	Wu et al. (2014)
	Simple coacervation	Gum arabic (GA)		39.8 ± 1.5	
	Simple coacervation	k-carrageenan (CG)		12.6 ± 0.6	
	Complex coacervation	MD/GA (25:75)		34.0 ± 2.5	
	Complex coacervation	GA/β-cyclodextrin (2:5)		29.4 ± 3.9	
Sulforaphane	Micelles	PCL–PEG–PCL copolymeric	Freeze-drying	87.1∗	Manjili et al. (2017)
Sulforaphane	Nano emulsification	mPEG-PCL	-	86.0 ± 1.6	Danafar et al. (2016)
Allyl isothiocyanate	Emulsification	Calcium alginate beads	-	82.8∗	Seo et al. (2012)
Allyl isothiocyanate	Complex coacervation	Chitosan/Gum Arabic	Spray drying	77.0∗	Ko et al. (2012b)
Allyl isothiocyanate	Emulsification	Gum arabic (GA)	Spray drying	84.7∗	Ko, Jeon & Park (2012a)
Allyl isothiocyanate	Complex coacervation	Gelatin/Gum arabic	Freeze-drying	94.2 ± 2.3	Wu et al. (2015)
Allyl isothiocyanate	Tannic acid cross-linked gelatin–gum arabic coacervate	Gelatin/Gum arabic	-	83.8 ± 2.8	Zhang et al. (2011)
Benzyl isothiocyanate	Inclusion complex	β-cyclodextrin	Freeze-drying	86.4 ± 2.6	Li et al. (2015a)
Benzyl isothiocyanate	Emulsion ionic gelation	Chitosan nanoparticles	Freeze-drying	64.7 ± 4.7	Uppal et al. (2018)

∗ Standard deviation not reported.

### 2.2 Chemical microencapsulation

#### 2.2.1 Coacervation

Coacervation is defined as the separation of colloidal systems into two liquid phases. Simple coacervation refers to a process where the polymer is salted out by the addition of electrolytes or an organic solvent, or by manipulating temperature (Castro-Rosas et al., 2017). Complex coacervation occurs due to attraction forces between oppositely charged polymers (Eghbala and Choudharyb, 2018). Anionic and cationic polymers interact with each other to form a polymer-rich phase called “complex coacervate” in equilibrium with the supernatant (Mishra, 2016). Temperature, core/wall material mass ratio, pH, and the mass ratio of the polymers determine the performance of microencapsulation by complex coacervation (Rudke et al., 2019).

Essential oils, vegetal extracts and natural bioactive compounds have been successfully microencapsulated by complex coacervation (Castro-Rosas et al., 2017). García-Saldaña et al. (2016) used complex coacervation for SFN microencapsulation using gelatin-gum Arabic and gelatin-pectin systems. They reported an extraction efficiency (EE) of 12.17 ± 0.10% and 17.91 ± 1.27%, respectively; and encapsulation yield (EY) of 85.13 ± 0.71% and 81.31 ± 0.02%, respectively. The difference between EE and EY was interpreted as sulforaphane loss. García-Saldaña et al. (2016) dissolved sulforaphane in the aqueous phase, what probably reduced EE given the low water-solubility of SFN. Wu, Zou, Mao, Huang & Liu (2014) encapsulated SFN through simple coacervation, using maltodextrin, gum Arabic and k-carrageenan, and also by complex coacervation using maltodextrin – gum Arabic and gum Arabic – β-cyclodextrin. After the chemical treatment, the microcapsules were subjected to spray-drying. The lowest encapsulation efficiency was obtained with k-carrageenan (12%); while the other systems gave significantly higher encapsulation efficiencies (30–40 %). Ko, Kin & Park (2012b) used complex coacervation for microencapsulation of allyl isothiocyanate with a chitosan-gum Arabic system, followed by spray-drying. The authors reported 77% encapsulation efficiency. Wu, Xue, Hou, Feng & Zhang (2015) encapsulated allyl isothiocyanate using a gelatin-gum Arabic system followed by freeze-drying, and found 94.2% encapsulation efficiency. Consequently, it could be speculated that dissolving SFN in the organic phase for its microencapsulation through complex coacervation would increase encapsulation efficiency, especially if followed by freeze-drying. This should be tested experimentally in the future.

#### 2.2.2 Molecular inclusion complex

Microencapsulation through molecular inclusion complex refers to the use of β-cyclodextrins to form a molecular complex stabilized by non-covalent interactions between the β-cyclodextrin and the compound to be encapsulated (Castro-Rosas et al., 2017). The core of β-cyclodextrins is hydrophobic, allowing the stabilization of hydrophobic molecules inside the complex, while the outer side is hydrophilic. This favors the interaction of the inclusion complex with the surroundings in aqueous solution.

Wu et al. (2014) studied microencapsulation of a SFN-rich broccoli seeds extract through spray drying and using different chemical encapsulation techniques; among them, inclusion complex. The latter technique resulted in a relatively low encapsulation efficiency (below 30%), in comparison with the other techniques assayed (between 34 and 70%). However, SFN inclusion complex exhibited the highest stability during storage at 35 °C during 28 days. This could probably compensate the lower yield obtained with inclusion complex as microencapsulation technique.

Martínez-Hernández et al. (2018) studied the effect of α-, β- and maltosyl-β-cyclodextrin for microencapsulating the glucoraphanin–sulforaphane system obtained from blanched broccoli juice. The authors found that β–cyclodextrin was the most suitable material for SFN microencapsulation, resulting in microcapsules that kept SFN content for 3 h at 22 °C. This modest result may relate to the use of water as solvent to obtain broccoli juice, considering that SFN is poorly water soluble. Probably in the juice there was no oil, and therefore SFN was more exposed. The results were much more promising for glucoraphanin, which is the highly water-soluble and stable precursor of SFN. Accordingly, the system proposed by Martínez-Hernández et al. (2018) does not represent a technological solution for the stabilization of SFN aiming at including this bioactive compound in elaborated foods.

Wu et al. (2010) investigated the stability of sulforaphane using inclusion complex with hydroxypropyl-β-cyclodextrin, and reported a retention of 97% for microencapsulated SFN, against 84% retention of free SFN in solution at 50 °C for 24 h. SFN stability at basic pH was considerably improved, with a retention of 92% for microencapsulated SFN at pH 8.0, in comparison with 58% retention of free SFN at the same pH. The authors reported much higher retention values than other works, even for free SFN, probably because they used an ethanolic extract form broccoli seeds, which most likely contained several compounds other than SFN, such as oil, which could have a protective effect on SFN. Then the high retention and improved stability reported by Wu et al. (2010) is partly explained by the extract composition, and it is not possible to ascribe these stabilizing effects only to hydroxypropyl-β-cyclodextrin microencapsulation.

Li et al. (2015a) studied microencapsulation of benzyl isothiocyanate through inclusion complex using β-cyclodextrin, focusing on the physical and chemical properties of microcapsules. This system would be suitable for stabilization and controlled release of benzyl isothiocyanate. Uppal et al. (2017) used ultrasound to assist benzyl isothiocyanate microencapsulation through inclusion complex, and reported a physicochemical characterization of the system. The inclusion complex kept antibacterial activity, and then the authors speculate that they also should keep the pharmacological properties. Yuan, Yao, Shen & Mao (2009) optimized the formation of benzyl- and phenyl isothiocyanates inclusion complexes using β-cyclodextrin, through response surface methodology. They concluded that temperature and mass ratio were the most significant factors that affect encapsulation.

Currently there is no study about optimization of SFN encapsulation in order to maximize EE or EY. This is a research line that should be further explored aiming at identifying the microencapsulation conditions that could be scaled up to industrial level, expanding the access of consumers to SFN.

#### 2.2.3 Nano emulsification and ionic gelation

An emulsion corresponds to a mixture of two immiscible liquids, one of them being dispersed as droplets (“disperse phase”) in the other (“continuous phase”). Depending on the droplet size, there are macro emulsions (above 100 μm), nano emulsions (20–100 μm), and micro emulsions (5–50 μm). Macro and nano emulsions are thermodynamically unstable, the first being opaque and the second, almost transparent. Micro emulsions are usually transparent and thermodynamically stable (McClements, 2010). Ionic gelation is an encapsulation method based on the ionic interaction between two oppositely charged polymers or between a polymer and a polyion. Alginate is an anionic polymer that forms ionic bonds with polyvalent cations, and it is mostly recommended for encapsulation of hydrophobic compounds (Benavides et al., 2016). Additionally, the internal gelation method (or emulsion ionic gelation) involves the formation of an emulsion where CaCO_3_ particles are dispersed in the alginate solution before the emulsification (Paques, Sagis, van Rijn & van der Linden, 2014).

Studies about encapsulation of SFN and other isothiocyanates through nano emulsification or ionic gelation are scarce. Danafar et al., (2017) encapsulated sulforaphane using monomethoxypoly (ethylene glycol)–poly (e-caprolactone) (mPEG–PCL), obtaining SFN micelles with an encapsulation efficiency of 86 ± 1.58%. Li et al. (2015b) prepared oil-in-water allyl isothiocyanate (AITC) nano emulsion by the method of emulsion inversion point, aiming at improving the aqueous stability of AITC. The nano emulsion showed superior protection against AITC degradation (78% remaining after 60 d at 30 °C), compared with protein nanoparticles as well as a non-encapsulated aqueous dispersion. Uppal et al. (2018) encapsulated benzyl isothiocyanate through emulsion ionic gelation using chitosan, and obtained an encapsulation efficiency of 64.7%. The nano capsules exhibited a maximum cumulative release of 77.8% in 144 h at pH 5.5 and 37 °C.

Ionic gelation has not been applied for SFN encapsulation, despite its suitability for hydrophobic compounds. Accordingly, this technique could probably help in SFN stabilization and most likely improve the results reported until now. This encapsulation method should be tested in the future.

### 2.3 Physical microencapsulation technique

Spray-drying is a low-cost process widely used for the microencapsulation of fragrances, oils, and flavors. The process is highly flexible since it allows variation of the microencapsulation matrix, and produces good quality particles (Mishra, 2016). Wu et al. (2014) investigated the effect of wall material on the microencapsulation of SFN using spray drying. The encapsulation efficiency fluctuated between 12.6% ± 0.6 (k-carrageenan) and 39.8% ± 2.5 (gum Arabic). These efficiencies are significantly lower than the efficiencies reported for microencapsulation of allyl isothiocyanate using Gum Arabic and spray drying, which range between 77% and 84.7% (Ko et al., 2012b). This relates with the higher thermo lability of SFN in comparison with other ITC. SFN degradation starts at temperature above 40 °C, while other ITC degrade above 70 °C (Van Eylluen et al., 2007). Accordingly, a disadvantage of spray drying is the relatively high operating temperatures, which impair the encapsulation efficiency of thermo labile compounds. EE of sulforaphane could probably be improved by using lower drying air temperature, considering that Wu et al. (2014) used inlet air temperature between 150 and 230 °C. The lowest inlet air temperature commonly used at industrial spray drying is 120 °C (Filková and Mujumdar, 1995). However, in spray-drying the contact time between the particles and the hot air is extremely short, and then moisture evaporation is assumed to occur instantaneously. Therefore the particle does not exceed the humid bulb temperature. Since SFN degradation starts at 40 °C (Mahn et al., 2016), some thermal degradation would probably be observed during spray drying. Accordingly, another drying method could be more adequate for SFN microencapsulation.

Freeze-drying exploits sublimation and is suitable for the encapsulation of aromas, water-soluble essences, drugs, and in general any heat sensitive material. The process requires a long dehydration period (Mishra, 2016), what in addition to the vacuum requirement increases the operating cost by around 4-fold in comparison with spray-drying (Ratti, 2001).

Manjili et al. (2017) investigated pharmacokinetics and delivery of SFN micelles obtained by freeze-drying. The authors reported an 87.1% encapsulation efficiency, significantly higher than the values obtained by spray-drying. Wu et al. (2015) studied the microencapsulation of allyl isothiocyanate intended for tomato preservation. They built the microcapsules using complex coacervation followed by freeze-drying, and reported encapsulation efficiencies between 68.6 ± 5.2 and 94.2 ± 2.3%. Li et al. (2015a) studied the microencapsulation of benzyl isothiocyanate using β-cyclodextrin and freeze-drying. They achieved 86.4 ± 2.6% encapsulation efficiency and proved β-cyclodextrin as a suitable wall material. Uppal et al. (2018) studied the microencapsulation of benzyl isothiocyanate using chitosan and freeze-drying, and reported a maximum encapsulation efficiency equal to 64.7 ± 4.7%.

In average freeze-drying yields higher encapsulation efficiency of SFN (87.1%) in comparison with vacuum drying (15%) and spray-drying (31%) (See Table 1). For allyl isothiocyanate freeze-drying gave a higher encapsulation efficiency (94.2%) in comparison with spray-drying (81% in average), a difference not as marked as for SFN. In the case of benzyl isothiocyanate, only freeze-drying data is reported, with an average encapsulation efficiency of 76%. Then, freeze-drying would be a much more efficient technology for microencapsulation of isothiocyanates and sulforaphane; however freeze-drying has the highest investment and operation cost. Then, the economic value of the encapsulated product would determine the feasibility of using freeze-drying instead of other dehydration method.

## 3 Effect of microencapsulation on sulforaphane stability

The degradation of most biological materials follows a first-order reaction kinetics (Eq. (1)). Therefore the stability of an encapsulated compound can be represented in this way. In Eqn (1), k is the rate constant, t is time, C_A0_ and C are the compound concentration at t = 0 and t = t, respectively. Eq. (2) describes the temperature dependence of k through the Arrhenius equation. Here, k_0_ is the rate constant at a reference temperature (T_ref_), T is temperature, E_A_ is activation energy, and R is the universal gas constant.

(1) $$\ln\frac{C}{C_{A0}}=kt$$

(2) $$\ln\frac{k}{k_{0}}=-\frac{E_{A}}{R}\times \left(\frac{1}{T_{ref}}-\frac{1}{T}\right)$$

Wu et al. (2013) studied thermal degradation kinetics of free SFN and the inclusion complex of SFN with hydroxypropyl-β-cyclodextrin at different pHs and at temperature between 60 and 90 °C. The authors demonstrated that pH has a significant effect on SFN stability and found that low pH reduces the SFN depletion rate. Also the authors reported that SFN microencapsulated with hydroxypropyl-β-cyclodextrin presents higher thermal stability than free SFN, with degradation kinetic constants being slightly higher for free SFN (see Table 2). The activation energies of both free and encapsulated SFN were higher at lower pH for all temperatures.

**Table 2:** Degradation kinetic constants for free and encapsulated sulforaphane at different temperatures and pH 6.0

Wall material	Storage temperature (°C)	Drying method	k (h^−1^)	Reference
Free	35	Spray-drying	0.019	Wu et al. (2014)
Gum Arabic (GA)			0.009	
GA + α-cyclodextrin			0.002	
MD + GA			0.004	
Maltodextrin (MD)			0.002	
Free	22	Freeze-drying	0.015	Fahey et al. (2017)
Free	37		0.094	
α-cyclodextrin	22		0.000	
α-cyclodextrin	50		0.004	
Free	60	Vacuum rotaevaporation	0.100	Wu et al. (2013)
Free	75		0.290	
Free	82		0.470	
Free	90		0.800	
Hydroxypropyl – β-cyclodextrin	60		0.070	
Hydroxypropyl – β-cyclodextrin	75		0.250	
Hydroxypropyl – β-cyclodextrin	82		0.390	
Hydroxypropyl – β-cyclodextrin	90		0.600	
Free	50	Vacuum rotaevaporation	0.007	Wu et al. (2010)
Hydroxypropyl – β-cyclodextrin	50		0.002

**Table 3:** Structure and sources of some isothiocyanates. Figure 3 shows the chemical structures of isothiocyanates.

Isothiocyanate	Precursor (Glucosinolate)	Food Sources
Phenethyl isothiocyanate	Gluconasturtiin	Watercress
Benzyl isothiocyanate	Glucotropaeolin	Cabbage, garden cress, Indian cress
Allyl isothiocyanate	Sinigrin	Broccoli, Brussels sprouts, cabbage, horseradish, kohlrabi, mustard, radish
Sulforaphane	Glucoraphanin	Broccoli, Brussels sprouts, cabbage, cauliflower, kale

Wu et al. (2014) investigated the storage stability of SFN microcapsules obtained using different wall materials. The systems were stored at 35 °C during 28 days, and Eqs. (1) and (2) were adjusted to estimate rate constants. Maltodextrin microencapsulation showed the highest stability (k = 0.002 [h^−1^]), while free SFN showed the lowest (k = 0.019 [h^−1^]) (See Table 2). This demonstrates the stabilizing effect of microencapsulation on SFN.

**Figure 2:**
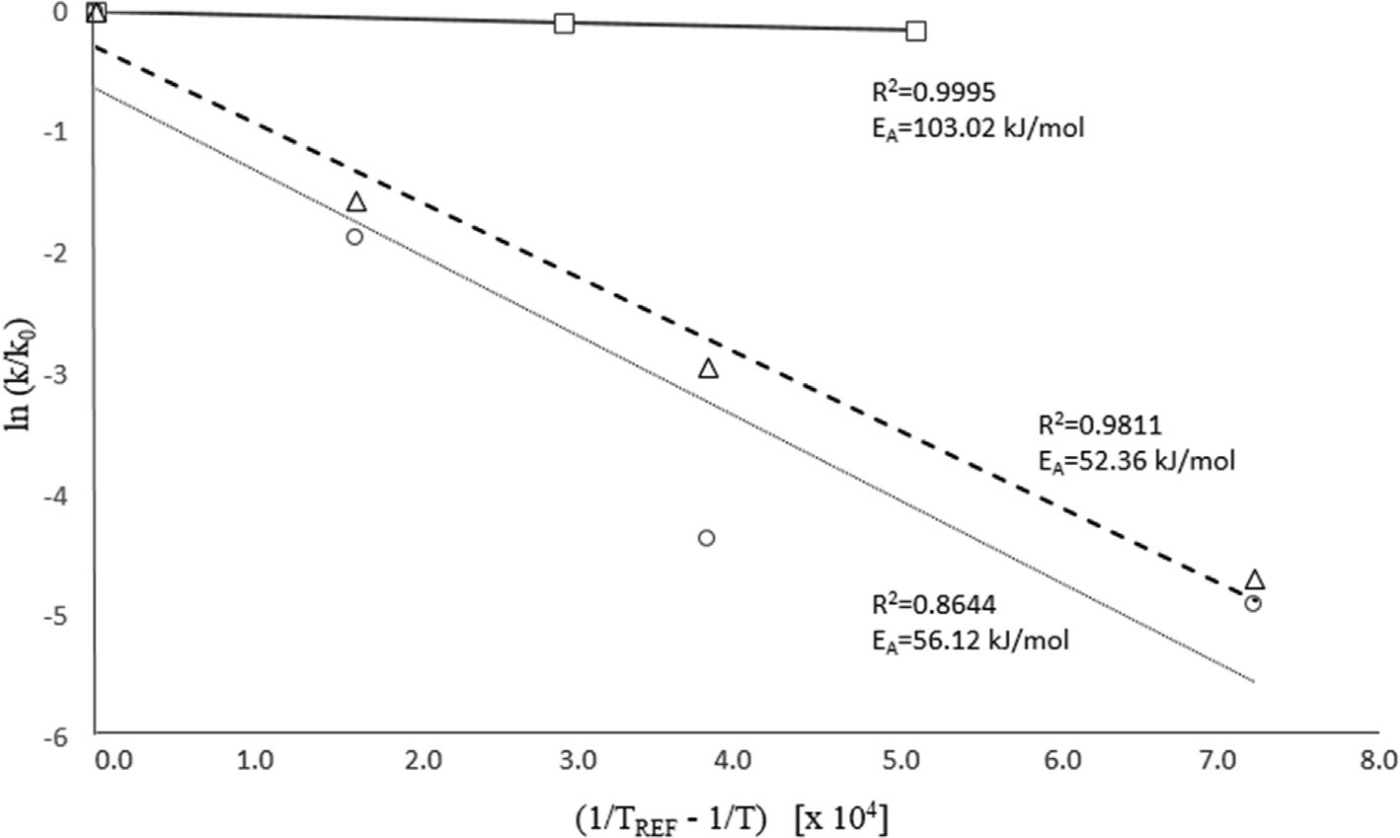
Arrhenius parameters for estimating the kinetic constants from the experimental data of Fahey et al. (2017). Free sulforaphane (○); Free sulforaphane in α-cyclodextrin solution (Δ); Sulforaphane microencapsulated in α-cyclodextrin (□).

Fahey et al. (2017) analyzed the stability of sulforaphane in different systems: aqueous solution of pure sulforaphane, aqueous solution of sulforaphane with α-cyclodextrin, and inclusion complex of SFN in α-cyclodextrin, at different temperatures. The authors did not inform degradation kinetic constants, but reported the results graphically. We acquired those data and adjusted to Eqs. (1) and (2), and estimated the rate constants and the activation energy, as shown in Figure 2. Table 2 shows the rate constants obtained from the results of Fahey et al. (2017). The decrease in the degradation rate constants of the encapsulated compound confirms the stabilizing effect of α-cyclodextrin on SFN. The E_A_ of free SFN (estimated from Fahey et al. (2017) data) was 56 [kJ/mol], agreeing with Mahn et al. (2016), who reported an E_A_ of 58 [kJ/mol] for SFN degradation during tray drying. The E_A_ of encapsulated SFN was 103.02 [kJ/mol], demonstrating that α-cyclodextrin microencapsulation significantly increased SFN stability.

**Figure 3:**
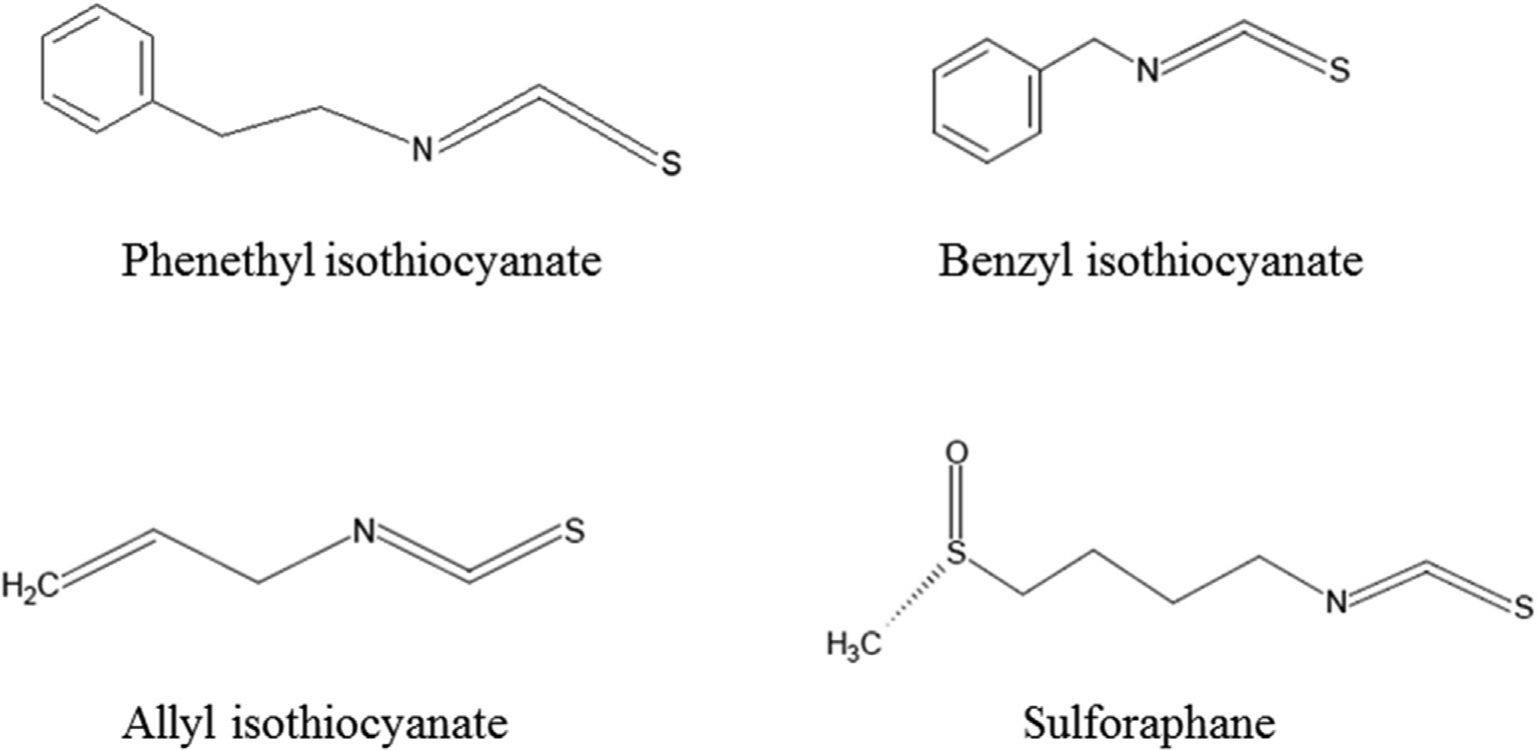
Chemical structures of isothiocyanates given in Table 3.

Wu et al. (2010) studied the stability of the inclusion complex of SFN and hydroxypropyl – β – cyclodextrin at 50 °C. The degradation kinetic constant was estimated from the data reported by Wu et al. (2010) resulting in 0.002 [h^−1^] (Table 2). This value agrees in order of magnitude with the constants obtained from Fahey et al. (2017) at the same temperature but using α-cyclodextrin as encapsulating agent. From those values it comes out that α-cyclodextrin has a higher stabilizing effect on SFN than hydroxypropyl – β – cyclodextrin. This agrees with the lower activation energy reported by Wu et al. (2013) for the inclusion complex of SFN with hydroxypropyl – β – cyclodextrin (75.1 kJ/mol) in comparison with the α-cyclodextrin complex reported by Fahey et al. (2017) (103.0 kJ/mol).

The degradation rate constant of free SFN reported by Fahey et al. (2017) differs significantly from that reported by Wu et al. (2014), probably because Fahey et al. (2017) did not use pure SFN but a SFN-rich extract obtained from broccoli sprouts, and then other compounds present in the extract could have acted as protecting agents thus decreasing the degradation rate in comparison with pure SFN.

## 4 Conclusions

The current literature shows that microencapsulation significantly improves sulforaphane stability. Some methods seem more adequate, especially ionic gelation and complex coacervation. Both methods are underexplored until now. The drying conditions used after chemical microencapsulation are extremely relevant, mainly because of thermal degradation of SFN. Optimization of SFN microencapsulation conditions has not been approached so far. This opens a relevant research line aiming at identifying the process conditions that could be used at industrial level. Finally, stability studies of microencapsulated sulforaphane in different systems are encouraged, since this information will help in designing SFN microencapsulation strategies that extend the industrial application of this promising health-promoting compound.

## Declarations

### Author contribution statement

All authors listed have significantly contributed to the development and the writing of this article.

### Funding statement

This work was funded by Vicerrectoría de Investigación, Desarrollo e Innovación through grant DICYT 081711MO, Universidad de Santiago de Chile.

### Competing interest statement

The authors declare no conflict of interest.

### Additional information

No additional information is available for this paper.
